# Detection and Characterization of Homologues of Human Hepatitis Viruses and Pegiviruses in Rodents and Bats in Vietnam

**DOI:** 10.3390/v10030102

**Published:** 2018-02-28

**Authors:** Dung Van Nguyen, Cuong Van Nguyen, David Bonsall, Tue Tri Ngo, Juan Carrique-Mas, Anh Hong Pham, Juliet E. Bryant, Guy Thwaites, Stephen Baker, Mark Woolhouse, Peter Simmonds

**Affiliations:** 1 Nuffield Department of Medicine, University of Oxford, Oxford OX1 3SY, UK; david.bonsall@ndm.ox.ac.uk (D.B.); peter.simmonds@ndm.ox.ac.uk (P.S.); 2 Wellcome Trust Major Overseas Programme, Oxford University Clinical Research Unit, Ho Chi Minh City 700000, Vietnam; cuongnv@oucru.org (C.V.N.); tuent@oucru.org (T.T.N.); jcarrique-mas@oucru.org (J.C.-M.); anhph@oucru.org (A.H.P.); gthwaites@oucru.org (G.T.); sbaker@oucru.org (S.B.); 3 Centre for Tropical Medicine and Global Health, Nuffield Department of Medicine, Oxford University, Oxford OX3 7FZ, UK; 4 Fondation Mérieux, Centre International de Recherche en Infectiologie (CIRI), 69365 Lyon CEDEX 07, France; juliet.bryant@fondation-merieux.org; 5 The London School of Hygiene & Tropical Medicine, London WC1E 7HT, UK; 6 Centre for Immunity, Infection and Evolution, University of Edinburgh, Edinburgh EH9 3FL, UK; mark.woolhouse@ed.ac.uk

**Keywords:** rodents, bats, pegiviruses, hepatitis viruses, homologues, Vietnam

## Abstract

Rodents and bats are now widely recognised as important sources of zoonotic virus infections in other mammals, including humans. Numerous surveys have expanded our knowledge of diverse viruses in a range of rodent and bat species, including their origins, evolution, and range of hosts. In this study of pegivirus and human hepatitis-related viruses, liver and serum samples from Vietnamese rodents and bats were examined by PCR and sequencing. Nucleic acids homologous to human hepatitis B, C, E viruses were detected in liver samples of 2 (1.3%) of 157 bats, 38 (8.1%), and 14 (3%) of 470 rodents, respectively. Hepacivirus-like viruses were frequently detected (42.7%) in the bamboo rat, *Rhizomys pruinosus*, while pegivirus RNA was only evident in 2 (0.3%) of 638 rodent serum samples. Complete or near-complete genome sequences of HBV, HEV and pegivirus homologues closely resembled those previously reported from rodents and bats. However, complete coding region sequences of the rodent hepacivirus-like viruses substantially diverged from all of the currently classified variants and potentially represent a new species in the *Hepacivirus* genus. Of the viruses identified, their routes of transmission and potential to establish zoonoses remain to be determined.

## 1. Introduction

Unlike many other communicable diseases, the burden of viral hepatitis has substantially increased over the last two decades to recently become the seventh leading cause of mortality worldwide. Viral hepatitis now causes more deaths than tuberculosis, AIDS or malaria each year. Hepatitis C virus (HCV) and hepatitis B virus (HBV) are responsible for >90% (96% in 2013) of viral hepatitis-related mortality and disability. As such, these hepatitis viruses are the targets of efforts to combat viral hepatitis [1], including HBV vaccination, development of HCV vaccines, and highly effective drugs. In contrast, hepatitis E virus (HEV) is endemic in many low-income countries [2] but usually causes self-limiting hepatitis. Infection with HEV occasionally results in liver failure [1].

HBV, HCV, and HEV are members of virus families *Hepadnaviridae*, *Flaviviridae*, and *Hepeviridae*, respectively. HBV has a partially double-stranded DNA genome with 4 overlapping open reading frames (ORFs) [3], whereas HCV and HEV have a single-stranded RNA genome [4,5]. While the origins of these human viruses are unknown, rodents and bats are putative reservoir hosts because they host a diverse array of *Hepadnaviridae* [6,7,8], *Hepeviridae* [7,9,10,11,12,13,14,15,16], and genera *Pegivirus* [17,18,19] and *Hepacivirus* [17,19,20] of the *Flaviviridae* family including homologues of the human hepatitis viruses under question. Among these, it is of concern that a bat hepadnavirus may possess the ability to infect human liver cells [6].

Several factors may contribute to the risk of zoonotic rodent or bat virus transmission. Rodent meat is a popular source of protein for human consumption in Vietnam, particularly in the Mekong Delta, where rats (*Rattus* spp. and *Bandicota indica*) are commonly trapped and sold live in wet markets [21]. The total annual consumption of rat meat in Vietnam is estimated at 3300–3600 tonnes [22]. Hoary bamboo rats (*Rhizomys pruinosus*) are additionally farmed for human consumption. Moreover, bat faeces collected from bat caves or farms is used as natural fertilizer (“guano”) in Vietnam. As rodents and bats are reservoirs or carriers of a significant number of zoonotic pathogens [23] and viruses with unknown zoonotic potential, there are health risks that are associated with exposure to these animals. However, a previous study [22] showed none of the surveyed rat catchers or processors were aware of infection risks from contact with live rats. Consequently, no precautions were taken for the handling of rodents.

In the search for viral diversity and zoonotic viruses, novel paramyxovirus and coronavirus in Vietnamese bats and rats were detected in a previous study [24]. Here, we report the detection pegivirus and human hepatitis-related viruses in these mammals.

## 2. Materials and Methods

### 2.1. Sample Collection

Rodent and bat samples were collected within the VIZIONS (Vietnam Initiative on Zoonotic Infections) framework [25] for the screening of zoonotic microorganisms [21,26,27,28].

**Rodent samples**. As it is important and essential to understand the risk associated with rodents, including those sold in the markets, a total of 435 rats purchased from markets in five of twelve provinces in the Mekong Delta during 2012–2015 and 82 farmed bamboo rats purchased from a market in Dak Lak in 2014–2015 were enrolled. In addition, 226 trapped rats were also included. Rat trapping was carried out in different locations (pig and poultry farms, rice fields, fruit groves, tropical forests, markets, slaughter-house) in the provinces of Dong Thap during March 2013 and Dak Lak in April 2014, as previously described [27]. Serum and liver samples were collected post-mortem. Species identification of rats was carried out on the basis of morphological characteristics and sequencing of a conserved housekeeping gene [27]. All of the samples were stored in sterile tubes with RNA later at −20 °C until nucleic acid extraction. Special precautions were taken to avoid cross-contamination.

**Bat samples**. A total of 157 bats were trapped at six sites in the provinces of Dong Nai (in Cat Tien National Park) and Quang Ngai in May 2013 using mist nets and harp traps as described [26]. Trapped bats were speciated according to morphology [29], and liver samples were collected and stored as described above for rats.

This study was approved by the People Committees of Dong Thap (No. 47/UBND-KTN, 23 January 2013) and Dak Lak (No. 5407/UBND-TH, 07 August 2013) provinces and the Oxford Tropical Research Ethics Committee (OxTREC) (No. 157-12, 10 September 2012) in the United Kingdom.

### 2.2. Nucleic Acid Extraction

RNA was manually extracted from 638 rodent serum samples using QIAamp Viral RNA Mini Kit (Qiagen, Manchester, UK) and following instructions from the manufacturer.

Liver samples from 157 bats and 470 rodents were subjected to nucleic acid extraction using AllPrep DNA/RNA Mini Kit (Qiagen, Manchester, UK). Briefly, about 30 mg of liver per sample was first lysed and homogenised using TissueLyser (Qiagen, Manchester, UK). The lysate was applied to an AllPrep DNA spin column for DNA to bind onto the column. Ethanol was added to the flow-through and RNA and bound to the membrane when the sample was passed through an RNeasy spin column. After washing steps, DNA and RNA was eluted separately in 50 μL of nuclease-free water. Extracted nucleic acid was used in screening for the targeted hepatitis viruses.

### 2.3. Screening of Hepatitis Viruses and Pegivirus

In order to minimize contamination, PCR mastermix preparation, and the addition of templates were carried under separated laminar flow hoods and lab spaces. All of the surfaces, tubes, and equipment were UV irradiated between each PCR. Reverse transcription using SuperScript III reverse transcriptase (Invitrogen, Paisley, UK) was performed according to the manufacturer’s instruction. Synthesized cDNA was screened for hepaciviruses and pegiviruses using a semi-nested PCR protocol with broad spectrum degenerate primers, which can detect all known hepaciviruses and pegiviruses. Amplification conditions (using GoTaq from Promega, Southampton, UK) included 95 °C for 3 min, and 30 cycles of denaturation (94 °C, 30 s), annealing (55 °C, 30 s) and elongation (72 °C, 30 s). Similarly, HEV was screened using broadly reactive primers targeting viral RNA-dependent RNA polymerase as described in Drexler et al., 2012 [11].

DNA extracted from liver samples was used for screening of HBV. Degenerate primers targeting a highly conserved region of the polymerase gene of sequences from all known HBV hosts were designed for a nested PCR protocol using the above amplification conditions. Primers for screening are listed in Table S3.

The length of the sequenced screening fragments (excluding primer sequences) of homologues of HBV, HEV, HCV, and pegivirus was 257, 284 and 360 nucleotides, respectively.

### 2.4. Complete Genome Sequencing

For rodent hepacivirus, HEV and pegivirus genomes, extracted RNA from representative positive samples was subjected to deep sequencing using an Illumina platform. Libraries were prepared from total extracted RNA using the NEBNext Ultra Directional Sequencing Kit (New England Biolabs, Hitchin, UK) with omission of actinomycin D, then pooled and sequenced on a HiSeq 4000 instrument (Illumina, Nr Saffron Walden, UK). Quality control and trimming of reads were performed before genome mapping using CLC Genomics Workbench (Qiagen Bioinformatics, Redwood City, CA, USA) with the default affine gap cost parameters. The closest related virus genomes (Genbank numbers KC815310, JX120573 and KJ950934 for hepacivirus, HEV and pegivirus, respectively) were used as templates for genome mapping. Additional primers were designed using the obtained contigs for gap fillings.

For bat HBV, primers were designed using sequences available from Genbank and the obtained sequences from screening. These primers amplified amplicons, with overlapping regions as presented in Table S4. All of these nested or hemi-nested PCR protocols used SuperScript III One-Step RT-PCR System with Platinum Taq DNA polymerase (Invitrogen, Paisley, UK) for RNA viruses or Q5 High-Fidelity DNA Polymerase (New England Biolabs, Hitchin, UK) for HBV in the first round PCR, according to instructions from the manufacturers. Q5 High-Fidelity DNA Polymerase was also used in the second round PCR.

### 2.5. Hepacivirus RNA Titer Measurement

Two real-time PCR primer sets (Table S5) in the 5′ untranslated region of bamboo rat hepaciviruses and the screening fragment of other rat hepaciviruses were designed using sequences available from this study. The targeted regions were amplified from positive samples and cloned into pGEM-T Easy Vector (Promega, Southampton, UK) for in vitro transcription, as previously described [30]. The obtained RNA transcripts were used to generate standard curves of the real-time PCR assays for measurement of rodent hepacivirus RNA titers using SuperScript III reverse transcriptase (Invitrogen, Paisley, UK) and PowerUp SYBR Green master mix (Thermo Fisher Scientific, Northumberland, UK).

### 2.6. Sequence Analysis

Sequences were imported into SSE (Simmonic Sequence Editor) [31] for the alignment and calculation of sequence distance values from reference sequences of known viruses from which sequence identities were inferred. Sequence distances instead of sequence identities in the regions used for classification of hepaciviruses and pegiviruses were presented to easily compare with the species p-distance thresholds set in the proposed update to the taxonomy of the genera *Hepacivirus* and *Pegivirus* [32]. Maximum-likelihood phylogenetic trees were reconstructed using the MEGA 7.0 software package [33] with 1000 bootstrap resamples. The best-fitting model for each sequence dataset (as shown in figure captions) was first determined and used for phylogenetic reconstruction. Sequences obtained in this study have been assigned the following GenBank accession numbers MG600410–MG600465.

## 3. Results

### 3.1. Detection of Hepatitis Viruses in Bat and Rodent Liver Samples

Nucleic acid that was extracted from liver samples of 157 bats (29 species; Table S1) and 470 rodents (six species) was screened for pegivirus and human hepatitis B, C, E viruses and their homologues (Table 1) by nested and semi-nested PCR assays with degenerate primers. Hepaciviruses were the most commonly detected (8.1% of rodent samples, from three species), followed by hepatitis E related virus (3% of rodent samples, from four species) while hepatitis B related viral DNA was only detectable in three bats (2 species). Most of the hepacivirus positive samples were from farmed hoary bamboo rats in Dak Lak province although the predominantly sampled rat species was *Rattus argentiventer*. Coinfection with hepacivirus and HEV was observed in a sample from *Rattus losea*. All liver samples from bats were negative for hepacivirus and hepatitis E related virus and no sample was positive for pegivirus.

### 3.2. Screening of Rodent Serum Samples for Hepacivirus and Pegivirus

Serum samples from 638 rodents (from eight species; Table S2) were screened for pegivirus and hepacivirus RNA simultaneously using the same primer set. This sample set included 30 bamboo rats and 335 other rats whose liver samples were screened for hepacivirus as above. Hepacivirus RNA was only detected in serum samples of 10 bamboo rats with positive liver samples. Pegivirus RNA was detected in two samples from *Rattus tanezumi*.

**Figure 1:**
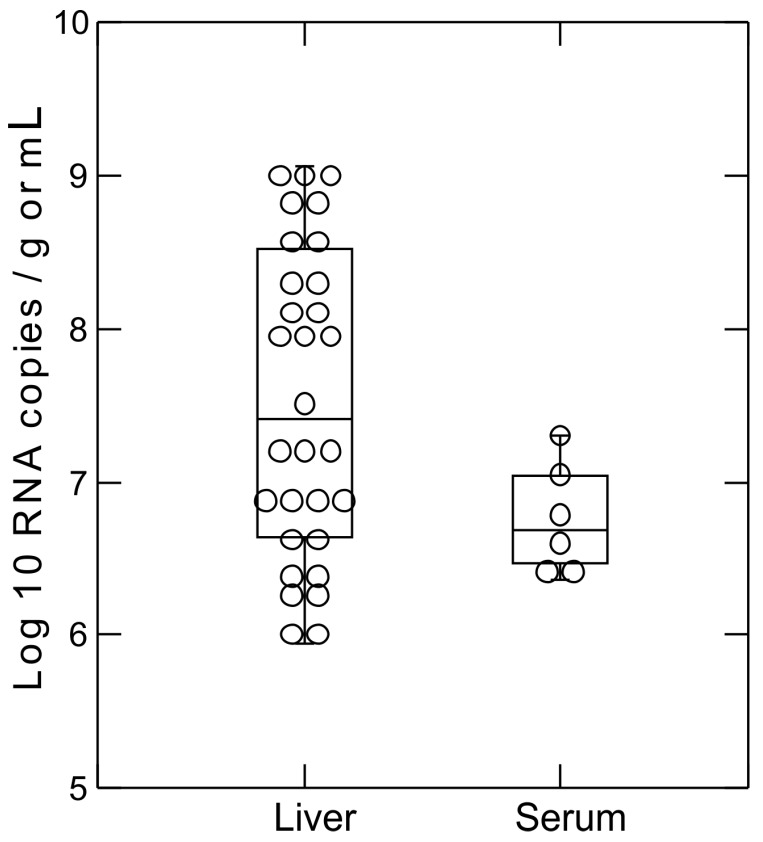
Box plot of rodent hepacivirus RNA concentration with each dot representing one sample.

Two real-time PCR assays specific for bamboo rat hepaciviruses, and other hepaciviruses were used to determine viral RNA concentration in the positive samples. The concentration of RNA (Figure 1) was high in both liver tissue (median, 3.35 × 10^7^ copies/gram; range, 0.9 × 10^5^–1.16 × 10^9^) and sera (median, 5.7 × 10^6^ copies/mL; range, 2.3 × 10^6^–2 × 10^7^).

### 3.3. Sequence and Phylogenetic Analysis

**Figure 2:**
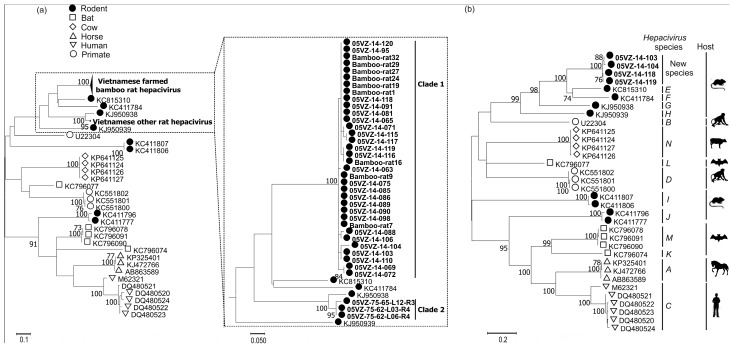
Maximum likelihood trees of (**a**) the screening fragment and (**b**) the amino acid region 1123–1566 of hepaciviruses using best-fitting models of rtREV+G+I and LG+G+I, respectively. The magnified box shows the two Vietnamese rodent hepacivirus clades. Labels for sequences obtained in this study are highlighted in bold. Bootstrap support values of ≥ 70% are shown. The trees were drawn to scale; bar, estimated number of substitutions per site.

Amplicons derived from PCR screening experiments were all successfully sequenced and complete or near complete genome sequences were determined for representatives of the viruses by PCR or deep sequencing. After exclusion of primers, the obtained screening sequences from each targeted virus clustered in one or two clades of those with high identity and were most closely related to sequences previously reported from rodents or bats from the US [17,18], China [7] and Vietnam [16]. Rodent hepacivirus sequences (360 nucleotides) formed two well supported clades (Figure 2a). Clade 1 included all of 35 sequences from *Rhizomys pruinosus* which shared 84.5–100% pairwise nucleotide identity while three sequences (nucleotide identity of 89–99%) from *Rattus losea* and *Rattus argentiventer* grouped in clade 2. These clades differed from each other by mean distances of 39.6% and 33.2% at nucleotide and amino acid levels, respectively. While the four Vietnamese bamboo rat hepacivirus genomes were highly similar to each other (<12% nucleotide and <3% amino acid distances in the complete coding region (cds)), they were remarkably different from the closest sequence (KC815310) with the corresponding distances of 40% and 36%, respectively (Table 2). The amino acid p-distances of the obtained hepacivirus sequences and KC815310 in the regions 1123–1566 and 2536–2959 (numbered relative to M62321) were 30% and 32%, respectively. The newly identified hepaciviruses therefore could be provisionally classified as members of a new hepacivirus species (Figure 2b) [32]. The other bamboo rat hepaciviruses in clade 1 may also belong to this new virus species on the basis of the high sequence identity in this clade. Similarly, although complete genome sequences were not determined for hepaciviruses in clade 2, they likely belong to species *Hepacivirus G* due to their low amino acid p-distances (7.6–8.4%) in the screening region to KJ950938. The 5’ untranslated region sequences of these hepaciviruses contain two miR-122 seed sites (CACUCC), which were located 51 nucleotides from each other, as consistent with the suspected hepatotropism of the viruses.

**Figure 3:**
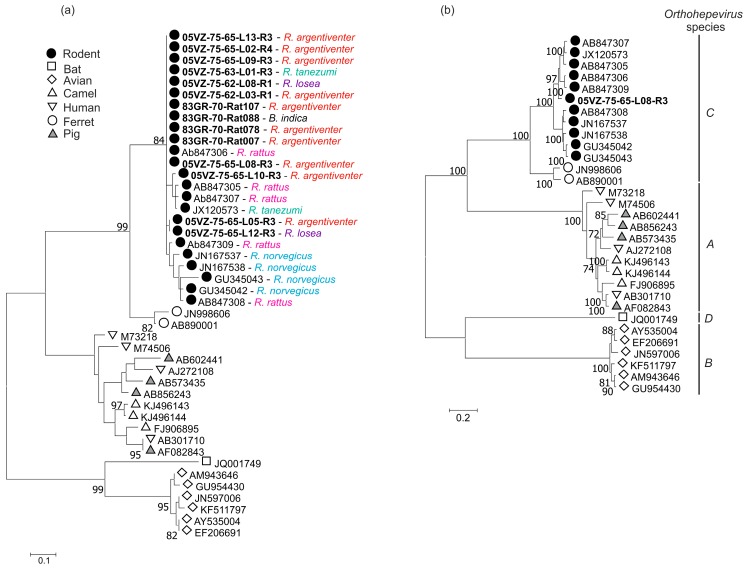
Maximum likelihood trees of (**a**) the screening fragment and (**b**) the ORF1 of hepeviruses using the best-fitting models of LG+G+I and LG+G+F, respectively. Rodent species are shown. Labels for sequences obtained in this study are highlighted in bold. Bootstrap support values of ≥ 70% are shown. The trees were drawn to scale; bar, estimated number of substitutions per site.

In contrast to host specificity of rodent hepaciviruses, the 15 HEV sequences (284 nucleotides) from four rodent species were 84.4–99.3% identical to each other and phylogenetically interspersed with each other and with reference sequences from other rat species (Figure 3a). The obtained complete genome of rat HEV comprised of 6960 nucleotides excluding the poly A tail. Its concatenated ORF1 and ORF2 differed by 6.8% (Table 2) at amino acid level to the closest match (JX120573) and these two sequences therefore belong to a same genotype in the species *Orthohepevirus C* (Figure 3b), according to the latest proposal for classification of hepeviruses [34].

**Figure 4:**
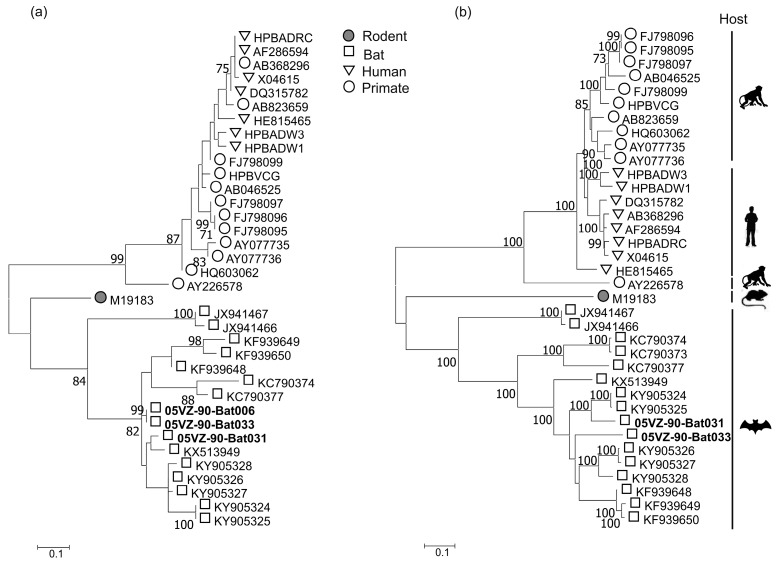
Maximum likelihood trees of (**a**) the screening fragment and (**b**) the polymerase of hepadnaviruses using the best-fitting models of JTT+G and JTT+G+I+F, respectively. Labels for sequences obtained in this study are highlighted in bold. Bootstrap support values of ≥ 70% are shown. The trees were drawn to scale; bar, estimated number of substitutions per site.

The three HBV variants (from bat species *Hipposideros pomona* and *Hipposideros larvatus*) clustered with known bat HBV sequences (Figure 4). The two bat HBV complete genome sequences comprised 3275 and 3302 nucleotides. As with other hepadnaviruses, the bat HBV genomes have four open reading frames (ORFs) encoding the surface (S), polymerase (P), core (C), and X proteins. A detailed comparison of the ORFs of these viruses with their closest sequences is shown in Table 2. Bat031 consistently showed greatest sequence identity to KY905324 in all 4 ORFs. In contrast, Bat033 shared highest identity to KY905328 in the P and S ORFs, but was more similar to KY905324 and KY905327 in the ORFs encoding for X and C, respectively.

**Figure 5:**
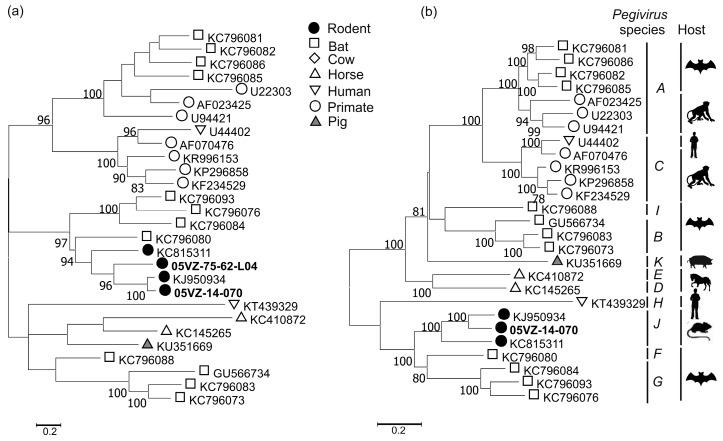
Maximum likelihood trees of (**a**) the screening fragment and (**b**) the region 888–1635 of pegiviruses using the best-fitting model of LG+G+I+F. Labels for sequences obtained in this study are highlighted in bold. Bootstrap support values of ≥ 70% are shown. The trees were drawn to scale; bar, estimated number of substitutions per site.

The two Vietnamese pegivirus sequences from *Rattus tanezumi* grouped with a sequence previously reported from *Rattus norvegicus* (Figure 5a). The amino acid p-distance between the obtained rodent pegivirus sequence and KJ950934 in the region 888–1635 (numbered relative to U22303) was 17.8% and the two viruses could be classified as members of species *Pegivirus J* (Figure 5b), according in the update to the taxonomy of the *Pegivirus* genus [32].

## 4. Discussion

The present study reports the findings of pegivirus and human hepatitis-related viruses in Vietnamese rodents and bats. The detection of hepacivirus, HEV homologue and pegivirus in a number of rodent species, and HBV homologue in *Hipposideros larvatus* indicates wider host ranges of these viruses. Whereas, the identified rat HEV and bat HBV showed relatively high sequence identity to previously characterized viruses infecting *Rattus rattus, Rattus tanezumi* and *Hipposideros pomona*, the rodent pegivirus and hepacivirus showed substantial sequence distances to their closest sequences and represent new pegivirus variants and a new hepacivirus species. This highlights the incomplete genetic characterization of these viruses. Thus far, only one complete genome and two complete coding sequences (including the one from this study) of rodent pegivirus are available on Genbank. More highly divergent hepacivirus and pegivirus sequences are expected to be discovered from rodents in future studies.

The absence of bat HEV, hepacivirus, pegivirus, and low detection frequency of bat HBV are consistent with their low prevalence (0–5%) reported in previous studies [6,7,11,19]. Contrastingly, the prevalence of hepacivirus in the farmed bamboo rats was unprecedentedly high (42.7%). The inbred nature of farmed bamboo rats that were investigated in this study may have contributed to susceptibility to infection and likelihood of persistence [35,36]. The existence of relatively homozygous individuals may play a key role in the maintenance of pathogens in a population [37].

The high prevalence of hepacivirus in bamboo rats also indicates the need to reconsider transmission routes of hepaciviruses. Among hepaciviruses, the transmission route of HCV has been relatively well studied, while those of other hepaciviruses are mostly speculative [38,39]. As a bloodborne virus, HCV is thought to be mainly transmitted through injections or blood transfusion. However, this does not explain how a range of divergent HCV strains have been maintained for centuries in some rural populations in central Africa and southeast Asia [40]. Equine hepacivirus has been shown to be transmittable via direct inoculation [41] and via vertical transmission [42]. Rodent hepacivirus may utilize a similar transmission route as experimental intravenous injection of the supernatants of homogenised liver tissues from infected rats lead to viraemia [43]. The high prevalence of hepacivirus in bamboo rats (in this study) and commensal *Rattus norvegicus* (23.3% in Firth et al. 2014 [18]) indicates other more efficient transmission routes may exist such as via saliva and bites, which are likely to occur in caged conditions with a density of 15–20 individuals/2 m^2^ cage. Infection experiments (i.e., exposure of hepacivirus negative bamboo rats to hepacivirus positive saliva) may elucidate transmission routes of the new hepacivirus.

The zoonotic potential of the detected viruses is unknown and requires further investigation. While the identified rodent hepaciviruses appear host species specific, four rodent species were infected with highly similar HEV homologues, which were phylogenetically interspersed, indicative of low host species specificity. This is a characteristic that may lead to their establishment and emergence in new hosts. Understanding the receptor usage for cell entry of HEV in rodents and other host species would potentially help predict the host range of the virus. Furthermore, a surrogate assay with pseudotyped viruses carrying surface/envelope proteins of the identified viruses may be useful in assessing their potential to infect human liver cells. Such an assay was used to show that a bat HBV could infect primary human hepatocytes [6].

In summary, this study demonstrated the wide circulation of diverse pegivirus and human hepatitis-related viruses in new rodent and bat species. The presented findings form a framework for future investigations of human transmission risk, now that the rodent and bat species infected with these viruses have been identified and the human contact groups are better defined (e.g., bamboo rat farmers, rat catchers, rat sellers, and bat farmers). The transmission routes of the identified viruses are to be determined.

## Figures and Tables

**Table 1:** Detection of hepatitis related viruses in rodent liver samples.

Species	Tested	Positive (%)		
		Hepacivirus	HEV	HBV
*Bandicota indica*	38	0	1 (2.6)	0
*Rattus argentiventer*	275	2 (0.7)	10 (3.6)	0
*Rattus losea*	19	1 (5.3)	2 (10.5)	0
*Rattus norvegicus*	39	0	0	0
*Rattus tanezumi*	17	0	1 (5.9)	0
*Rhizomys pruinosus*	82	35 (42.7)	0	0
Total	470	38 (8.1)	14 (3)	0

**Table 2:** Nucleotide and amino acid identities of Vietnamese rodent and bat virus sequences to the closest matches.

Virus	Sequence	Compared Region	Highest Nucleotide Identity (%)	Highest Amino Acid Identity (%)	Closest Match
Rodent hepacivirus	05VZ-14-103	Complete cds	59.7	63.3	KC815310
	05VZ-14-104	Complete cds	59.7	63.2	KC815310
	05VZ-14-118	Complete cds	59.5	63.3	KC815310
	05VZ-14-119	Complete cds	59.5	63.3	KC815310
Rodent HEV	05VZ-75-65-L08-R3	ORF1 + ORF2	81.5	93.2	JX120573
Bat HBV	Bat031	*P* gene	89	87	KY905324
		*S* gene	91	94	KY905324
		*X* gene	92.4	86	KY905324
		*C* gene	90	96.3	KY905324
	Bat033	*P* gene	82.5	80	KY905328
		*S* gene	87	84	KY905328
		*X* gene	90	80	KY905324
		*C* gene	89	97.2	KY905327
Rodent pegivirus	05VZ-14-070	Complete cds	65.2	65.5	KJ950934

## Supplementary Materials

The following are available online at http://www.mdpi.com/1999-4915/10/3/102/s1.

## Appendix A

The VIZIONS Consortium members are

Oxford University Clinical Research Unit (alphabetic order by sur-name): Bach Tuan Kiet, Stephen Baker, Alessandra Berto, Maciej F. Boni, Juliet E Bryant, Bui Duc Phu, James I Campbell, Juan Carrique-Mas, Dang Manh Hung, Dang Thao Huong, Dang Tram Oanh, Jeremy N. Day, Dinh Van Tan, H. Rogier van Doorn, Duong An Han, Jeremy J Farrar, Hau Thi Thu Trang, Ho Dang Trung Nghia, Hoang Bao Long, Hoang Van Duong, Huynh Thi Kim Thu, Lam Chi Cuong, Le Manh Hung, Le Thanh Phuong, Le Thi Phuc, Le Thi Phuong, Le Xuan Luat, Luu Thi Thu Ha, Ly Van Chuong, Mai Thi Phuoc Loan, Behzad Nadjm, Ngo Thanh Bao, Ngo Thi Hoa, Ngo Tri Tue, Nguyen Canh Tu, Nguyen Dac Thuan, Nguyen Dong, Nguyen Khac Chuyen, Nguyen Ngoc An, Nguyen Ngoc Vinh, Nguyen Quoc Hung, Nguyen Thanh Dung, Nguyen Thanh Minh, Nguyen Thi Binh, Nguyen Thi Hong Tham, Nguyen Thi Hong Tien, Nguyen Thi Kim Chuc, Nguyen Thi Le Ngoc, Nguyen Thi Lien Ha, Nguyen Thi Nam Lien, Nguyen Thi Ngoc Diep, Nguyen Thi Nhung, Nguyen Thi Song Chau, Nguyen Thi Yen Chi, Nguyen Thieu Trinh, Nguyen Thu Van, Nguyen Van Cuong, Nguyen Van Hung, Nguyen Van Kinh, Nguyen Van Minh Hoang, Nguyen Van My, Nguyen Van Thang, Nguyen Van Thanh, Nguyen Van Vinh Chau, Nguyen Van Xang, Pham Ha My, Pham Hong Anh, Pham Thi Minh Khoa, Pham Thi Thanh Tam, Pham Van Lao, Pham Van Minh, Phan Van Be Bay, Maia A Rabaa, Motiur Rahman, Corinne Thompson, Guy Thwaites, Ta Thi Dieu Ngan, Tran Do Hoang Nhu, Tran Hoang Minh Chau, Tran Khanh Toan, Tran My Phuc, Tran Thi Kim Hong, Tran Thi Ngoc Dung, Tran Thi Thanh Thanh, Tran Thi Thuy Minh, Tran Thua Nguyen, Tran Tinh Hien, Trinh Quang Tri, Vo Be Hien, Vo Nhut Tai, Vo Quoc Cuong, Voong Vinh Phat, Vu Thi Lan Huong, Vu Thi Ty Hang, Heiman Wertheim. Centre for Immunity, Infection and Evolution, University of Edinburgh (alpha-betic order by surname): Carlijn Bogaardt, Liam Brierley, Margo Chase-Topping, Al Ivens, Lu Lu, Dung Nguyen, Andrew Rambaut, Peter Simmonds, Mark Woolhouse. The Wellcome Trust Sanger Institute, Hinxton, United Kingdom (alphabetic order by surname): Matthew Cotten, Bas B. Oude Munnink, Paul Kellam, My Vu Tra Phan. Laboratory of Experimental Virology, Department of Medical Microbiology, Center for Infection and Immunity Amsterdam (CINIMA), Academic Medical Center of the University of Amsterdam, Amsterdam, The Netherlands (alphabetic order by surname): Lia van der Hoek, Martin Deijs, Maarten F. Jebbink, Seyed Mohammad Jazaeri Farsani. Metabiota, California, United States of America (alphabetic order by surname): Karen Saylors, Nathan Wolfe.
