# Supplementary material for: Detection and Characterization of Homologues of Human Hepatitis Viruses and Pegiviruses in Rodents and Bats in Vietnam
Source: Viruses. 2018 Feb 28;10(3):102. doi: 10.3390/v10030102 (PMC5869495; doi:10.3390/v10030102)
Supplement: Supplementary file 1 [file viruses-10-00102-s001.docx]

Table S1. Number of liver samples by bat species

| **Species** | **Number of samples** |
| --- | --- |
| *Cynopterus sphinx* | 14 |
| *Glischopus bucephalus* | 2 |
| *Hipposideros cineracea* | 1 |
| *Hipposideros armiger* | 6 |
| *Hipposideros cineracea* | 3 |
| *Hipposideros galeritus* | 4 |
| *Hipposideros larvatus* | 7 |
| *Hipposideros pomona* | 3 |
| *Kerivoula cf. hardwicki* | 4 |
| *Kerivoula kachinensis* | 2 |
| *Megaderma spasma* | 4 |
| *Megaderma lyra* | 1 |
| *Megaerops niphanae* | 3 |
| *Murina annamitica* | 1 |
| *Murina cyclotis* | 1 |
| *Murina eleryi* | 1 |
| *Murina walstoni* | 1 |
| *Myotis muricola* | 10 |
| *Myotis rosetti* | 6 |
| *Pipistrellus sp.* | 1 |
| *Rhinolophus acuminatus* | 25 |
| *Rhinolophus affinis* | 23 |
| *Rhinolophus cf. chaseli* | 3 |
| *Rhinolophus cf. lepidus* | 2 |
| *Rhinolophus luctus* | 1 |
| *Rhinolophus pusillus* | 12 |
| *Rhinolophus sinicus* | 9 |
| *Scotomanes ornatus* | 1 |
| *Scotophilus heathi* | 6 |
| **Total** | **157** |

Table S2. Number serum samples by rodent species

| **Species** | **Number of samples** |
| --- | --- |
| *Bandicota indica* | 73 |
| *Rattus argentiventer* | 292 |
| *Rattus exulans* | 76 |
| *Rattus losea* | 15 |
| *Rattus nitidus* | 25 |
| *Rattus norvegicus* | 40 |
| *Rattus tanezumi* | 87 |
| *Rhizomys pruinosus* | 30 |
| **Total** | **638** |

Table S3. Primers for screening

| **Target** | **Primer^*^** | **Sequence (5' - 3')** |
| --- | --- | --- |
| Hepaciviruses, pegiviruses | Flavi_4152s | GACGTIRTSATITGYGAYGA |
|  | Flavi_4569oa | AGTCRAARTTICCDGWRTAICC |
|  | Flavi_4549ia | CCIGTCATIAGRGCRTCIGT |
| Hepeviruses | HEV_4228s | ACYTTYTGTGCYYTITTTGGTCCITGGTT |
|  | HEV_R4598oa | GCCATGTTCCAGAYGGTGTTCCA |
|  | HEV_R4565ia | CCGGGTTCRCCIGAGTGTTTCTTCCA |
| Hepadnaviruses | HBV_266os | GTGGTGGAYTTCTCWCARTT |
|  | HBV_763oa | CCCCAAWACCANRTCATCCATA |
|  | HBV_386is | GATGTRTCTGCGGCGTTYTATC |
|  | HBV_687ia | CTAGTAAAYTGAGCCARGAGAAA |

^*^s, sense; os, outer sense; is, inner sense; ia, inner antisense; oa, outer antisense

Table S4. Primers for amplification of complete genome of HBV

| **Region** | **Primer** | **Sequence** |
| --- | --- | --- |
|  | HBV_1727os | GGTGGCTTTGGGGTATGGAC |
|  | HBV_324oa | GGGACAKACAGGAAGCAAGC |
|  | HBV_1795is | CTGCCTGTTGATTTCTTCCCG |
|  | HBV_274ia | TGAGGCATAGCAGCAGGACT |
|  | HBV_337os | GGAACCACCATGAGATGCAAC |
|  | HBV_2267oa | GGHGATTGAGATCKGCGTC |
|  | HBV_367is | GTCTCTGCGGACGAGATAACCTC |
|  | HBV_2252ia | CGTCKGCGAGGAGAGGGA |

Table S5. Real-time PCR primers for measurement of rodent hepacivirus RNA levels

| **Target** | **Primer** | **Sequence** |
| --- | --- | --- |
| Bamboo rat hepacivirus | Hepaci_199s | GGGCTAACGTGAACTACC |
|  | Hepaci_301a | GTACCATAAAGGCGGCTAC |
| Other rat hepacivirus | Hepaci_4136s | GCAGGCATCTTATCTTCCAGAC |
|  | Hepaci_4242a | GATGTCCTTGCCCCTGTAGT |
